# Behaviours that challenge in children with intellectual disability: systematic review and meta-analysis of pharmacological and non-pharmacological interventions

**DOI:** 10.1192/bjo.2025.10871

**Published:** 2025-10-27

**Authors:** Valerie Lye, Angela Hassiotis, Amanda Timmerman, Sohil Alqazlan, Elizaveta Dimitrova, Borbala Vegh, Vaso Totsika

**Affiliations:** Division of Psychiatry, University College London, London, UK; Special Education Department, College of Education, Imam Mohammad Ibn Saud Islamic University (IMSIU), Riyadh, Kingdom of Saudi Arabia

**Keywords:** Intellectual disability, challenging behaviour, children and adolescents, interventions, meta-analysis

## Abstract

**Background:**

Behaviours that challenge are highly prevalent in children with an intellectual disability and can be detrimental to their quality of life and opportunities.

**Aims:**

The systematic review aimed to investigate the effectiveness of current interventions in reducing behaviours that challenge in children with an intellectual disability (≤18 years-old).

**Method:**

We searched five databases (PsychINFO, MEDLINE, Embase, Web of Science and CINAHL) on 26 April 2022 and 1 July 2024, and identified 18 randomised controlled trials (1443 participants) eligible for inclusion since 2014 – 11 investigated non-pharmacological and 9 investigated pharmacological interventions. Risk of bias was assessed using the Cochrane Risk of Bias 2 tool.

**Results:**

Non-pharmacological interventions (mostly psychosocial) were significantly effective (Hedges’ *g* = −0.20; 95% CI [−0.35, −0.05]), whereas pharmacological interventions (including a wide range of drug classes and substances) were not (*g* = 0.03; 95% CI [−0.17, 0.24]). Studies using the Child Behaviour Checklist reported significant reductions (*g* = −0.18; 95% CI [−0.34, −0.02]), whereas studies using the Aberrant Behaviour Checklist did not (*g* = 0.04; 95% CI [−0.16, 0.25]). A random-effects meta-analysis indicated no overall significant reduction in behaviours that challenge (*g* = −0.12; 95% CI [−0.24, 0.00]).

**Conclusions:**

It is important to note that most studies included were conducted in Western countries and had small sample sizes, and findings may be due to the outcome measures used. Findings support current recommendations that non-pharmacological interventions should be first-line treatment for behaviours that challenge in this population. Evidence highlighted the need for better quality, adequately powered randomised controlled trials.

Intellectual disability is a developmental disability characterised by significant limitations in intellectual functioning (indicated by an IQ of less than 70) and adaptive behaviours (such as conceptual, social and practical skills used in daily life) that are evident in the developmental period.^1^

Behaviours that challenge or challenging behaviours are highly prevalent in intellectual disability, mostly due to difficulties experienced with communication, high prevalence of co-morbid mental health problems and low levels of meaningful activity.^2^ As a term, behaviours that challenge refers to phenotypically diverse behaviours, such as aggression towards the environment or others, self-injury and destructive behaviours. Behaviours that challenge are defined as such through their impact on the environment and/or the person as ‘behaviours of such an intensity, frequency or duration that the physical safety of the person or others is likely to be placed in serious jeopardy, or behaviour which is likely to seriously limit the use of, or result in the person being denied access to, ordinary community facilities’.^3^ Recent meta-analytic evidence indicates that behaviours that challenge are present in approximately 38 to 49% of children and adolescents with intellectual disabilities, depending on measurement, i.e. lower prevalence estimates were found in studies using the Developmental Behaviour Checklist (DBC) while the higher estimate was found when using the Child Behaviour Checklist (CBCL).^4^ Regardless of prevalence variation, it is evident that behaviours that challenge are highly prevalent, affecting up to half of children with intellectual disabilities.

Due to the nature and definition of behaviours that challenge, their measurement is complex.^5^ Standardised measures of behaviours that challenge, whether developed specifically for use with children with intellectual disabilities (e.g. the DBC^6^) or validated for use in this population (e.g. CBCL^7^), are important for a reliable and valid assessment of these behaviours.

Importantly, behaviours that challenge can be damaging to the quality of life of children and adolescents with intellectual disabilities, and people around them. It can lead to the exclusion of individuals from their local communities, make it difficult to form and maintain relationships, increase the risk of abuse and neglect and increase the risk of overmedication^8^ and caregiver burnout.^9^ Behaviours that challenge are associated with high costs for society and require large amounts of mental health resources.^10,11^ As a result, interventions to reduce the burden of behaviours that challenge are vital.

In the UK, the National Institute for Health and Care Excellence (NICE)^12^ conducted comprehensive systematic reviews and meta-analyses on the effectiveness of different types of interventions for behaviours that challenge in adults and children with intellectual disabilities. NICE considered evidence from randomised controlled trials (RCTs) published up to October 2014 and concluded that pharmacological interventions were effective in reducing behaviours that challenge in children and young people with intellectual disabilities. Effective pharmacological interventions included antipsychotics, such as risperidone (standardised mean difference, SMD = −1.07 [−1.31, −0.83]), aripiprazole (SMD = −0.64 [−0.91, −0.36]) and olanzapine (SMD = −1.40 [−2.73, −0.08]); antipsychotics plus anticonvulsants, such as topiramate plus risperidone (SMD = −1.88 [−2.63, −1.12]); and, likely, antioxidants such as N-acetylcysteine (SMD = −0.70 [−1.46, 0.05]). Inconclusive evidence was found in studies reporting on the use of anticonvulsants, such as valproate (SMD = −0.06 [−0.75, 0.63]) and biomedical interventions, such as omega-3 (SMD = 0.37 [−0.79, 1.53]) and ginkgo biloba (SMD = 0.10 [−0.47, 0.67]).

For non-pharmacological interventions, NICE included studies investigating environmental changes (including the physical and social environment) and a broad range of psychosocial interventions. NICE found that structured activity (SMD = −0.72 [−1.52, 0.08] to −1.21 [−2.06, −0.36]), behavioural parent training methods (SMD = −0.41 [−0.58, −0.24]), behaviour therapy (SMD = −0.47 [−0.98, 0.04]) and cognitive behavioural intervention (SMD = −0.24 [−0.63, 0.15]) were effective non-pharmacological interventions for behaviours that challenge in adults and children with intellectual disabilities. Sensory interventions were not effective (SMD = 1.42 [0.95, 1.88] to 2.22 [1.68, 2.75]).

Although the NICE reviews were comprehensive, the quality of RCTs included in the reviews were mostly classified as ‘low’ or ‘very low’. More recent meta-analyses (i.e. after October 2014) have focused on non-pharmacological interventions^9^ but did not examine their effectiveness for children with intellectual disabilities. Meta-analyses on mixed-age populations^13–19^ do not tend to review evidence separately for children and adolescents, except for Heyvaert et al^16^ who identified 11 studies including participants with intellectual disabilities below the age of 18 years. Heyvaert and colleagues^16^ found an SMD of 0.56 (s.e. 0.07; from a random-effects meta-analysis) suggesting moderate intervention effectiveness in reducing behaviours that challenge among children and adolescents with intellectual disabilities. The SMD referred to all types of interventions and the authors did not examine different types of interventions separately for this age group.^16^ It is worth noting that the Heyvaert et al^16^ meta-analysis included all types of study designs, not just RCTs. A more recent systematic review by O’Regan and colleagues^20^ investigated interventions to reduce behaviours that challenge among children with intellectual disabilities, but did not conduct a meta-analysis and similarly to Heyvaert,^16^ included all types of study designs, not just RCTs.

Overall, meta-analytic evidence is limited to studies before 2014 while evidence about non-pharmacological interventions is not specific to children and adolescents with intellectual disabilities. It is important to investigate children and adolescents separately from adults, as recent meta-analyses have shown that mental health symptoms and behaviours that challenge present differently in the two groups.^21^ Given the well-documented impact of treatments and behaviours of children and adolescents with intellectual disabilities, as well as the costs to services and society,^12^ we must determine whether current interventions are effective in reducing behaviours that challenge in this population.

Therefore, the primary objective of the present review was to provide a synthesis of recent evidence (after 2014) on intervention effectiveness in children and adolescents with intellectual disabilities and behaviours that challenge. We searched for studies published since October 2014, to identify studies published after NICE’s^12^ systematic reviews and meta-analyses.

In addition to overall intervention effectiveness, the present review aimed to investigate any association with intervention characteristics (type of intervention, i.e. pharmacological, non-pharmacological, and combined pharmacological and non-pharmacological interventions; and type of non-pharmacological intervention, i.e. psychosocial, physical activity, diet and occupational therapy) and the type of measurement scale used for assessing behaviours that challenge. Subgroup analyses were planned to align with the subgroup analyses done by NICE,^12^ where interventions that shared a broad key mechanism feature (e.g. were all medications) were grouped together. We did not have evidence that there would be enough studies within subgroups of interventions to plan further subgroup analyses (e.g. for specific pharmacological agents).

To ensure robustness, the present review only considered evidence from RCTs, as the currently recognised gold standard of effectiveness evidence, and studies that used well-validated measures of behaviours that challenge.

## Method

### Protocol

The review was pre-registered with PROSPERO on 11 April 2022 (registration number: CRD42022323529; https://www.crd.york.ac.uk/prospero/display_record.php?ID=CRD42022323529) and conducted following PRISMA guidelines^22^ (Supplementary Table S1 available at https://doi.org/10.1192/bjo.2025.10871).

### Search strategy

The following electronic bibliographical databases were searched: PsychINFO, MEDLINE, Embase, Web of Science and CINAHL. Searches were conducted using the following key words: (intellectual disab* OR learning disab*) AND (DBC* OR ABC* OR BPI* OR CBCL* OR SDQ* OR challenging behavi*) AND (random* controlled trial*). The last search was conducted on 1 July 2024. The full search strategies for all databases can be found in Table 1 and Supplementary Table S2. Additional search strategies included backwards and forwards reference searching of eligible studies and relevant review articles, using Google Scholar and Web of Science.

**Table 1:** Full search strategies for CINAHL Plus

Database	Full Search Strategy
CINAHL Plus	(learning disab* or learning difficult* or learning impair* or intellectual* disab* or intellectual* impair* or borderline intellectual* or development* disab* or development* disorder* or developmental delay or development* impair* or intellectual developmental disorder or mental* deficien* or mental* retard* or mental* handicap* or mental* disab* or mental* impair* or mental* challenged or mental* subnormal* or mental* sub-normal* or subaverage intelligence or subnormal or cognitive delay ) AND ( Developmental Behavi* Checklist* or DBC* or Aberrant Behavi* Checklist* or ABC* or Behavi* Problem* Inventory or BPI* or BPI-S or Child Behavi* Checklist* or CBCL* or “Strength* and Difficult* Questionnaire*” or SDQ* or challenging behavi* or maladaptive behavi* or aberrant behavi* or problem behavi* or behavi* problem* or behav* challenging or behav* disrupt* or behav* disturb* or behav* problem* or aggress* or destructive behavi* or property destruction or disruptive behavi* or stereotyp* or stereotyped behavi* or Repetitive behave* or self-injur* or self-injurious behavi* or SIB or self-harm or self hurt* or self wound* or ruminat* or Self damage* or self destruct* or self mutilat* or self violen* or parasuicid* or para-suicid* ) AND ( randomi* controlled trial* or RCT* or Clinical trial* or crossover or “cross over” or single* blind* or doubl* blind* or tripl* blind* or singleblind* or doubleblind* or tripleblind* or placebo* or random*)

### Inclusion and exclusion criteria

This review included studies (a) where ≥50% of participants were reported as having (i) an intellectual disability (mild, moderate, severe or profound) through diagnostic assessment, administrative eligibility (e.g. participants eligible for intellectual disability services) or parent/carer report, or (ii) a diagnosis of global developmental delay, or (iii) borderline intellectual functioning (BIF), defined by an IQ score between 70 and 85; (b) with any co-presenting mental or physical health problems, including co-representing neurodevelopmental conditions (e.g. autism, ADHD); (c) (i) where ≥75% of participants were under 18 years of age or the mean age of participants was under 18 years with a maximum age not exceeding 30 years, or (ii) data were presented separately for those under 18 years of age; (d) that used any type of comparators; (e) with an RCT; (f) with interventions that occurred in all settings (e.g. school, home, clinic, community); (g) with any type of intervention agent (e.g. professional, parent, teacher); (h) published in any language; (i) published after October 2014. Under pharmacological interventions, we considered any interventions using psychotropic medication or any other pharmaceutical or chemical agent. Non-pharmacological interventions encompassed all other types of interventions that were not pharmacological. We also included studies using combined pharmacological and non-pharmacological interventions. Studies were considered eligible for inclusion if they had measured behaviours that challenge as a primary OR secondary outcome using any of five pre-specified scales: the DBC/DBC-2^23^, the Aberrant Behaviour Checklist (ABC^24^), the Behaviour Problems Inventory (BPI/BPI-S^25^), the CBCL^7^ and the Strengths and Difficulties Questionnaire (SDQ^26,27^).

This review excluded studies with participants with primary neurodevelopmental conditions other than intellectual disability (e.g. autism, ADHD) without a co-presenting intellectual disability or an IQ score that placed them at risk of BIF and an unspecified developmental delay.

### Study selection

Eligible studies were independently screened by at least two reviewers. In stage one, the first reviewer (V.L.) screened the titles and abstracts of all returned records while additional reviewers (A.T., E.D., B.V. and S.A.) independently screened a random 20% of titles and abstracts (*n* = 714). Inter-rater reliability was excellent, with a Cohen’s kappa (*k*) of 0.77. In stage two, all full-text articles (*n* = 147) were reviewed by two reviewers working independently. Inter-rater reliability was excellent (*k* = 0.80). Any disagreements between the reviewers were resolved through discussions including a third reviewer (V.T.). Selected studies and decisions were recorded using the Covidence systematic review software (Veritas Health Innovation; Melbourne, Australia; https://www.covidence.org).

**Fig. 1:**
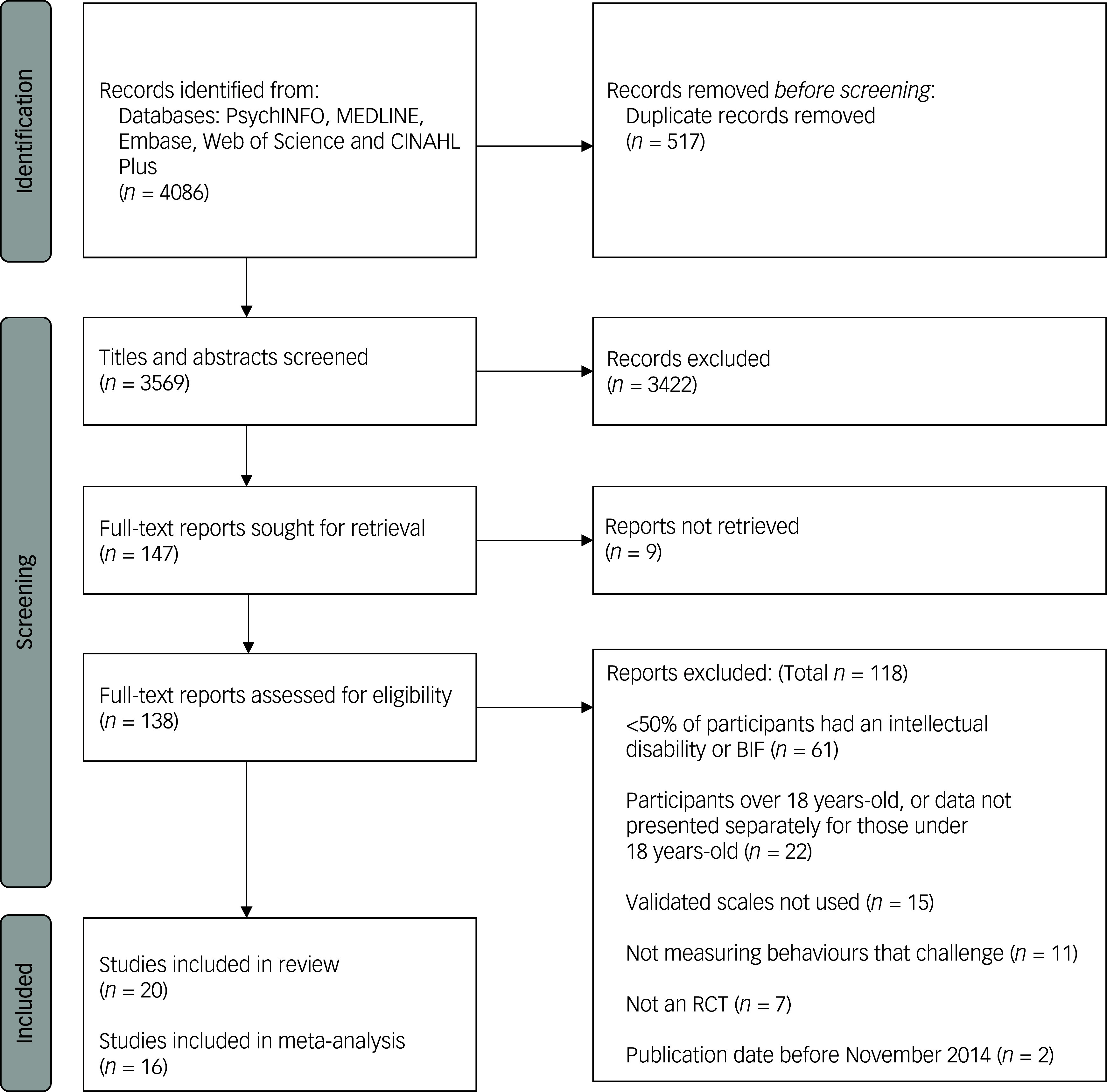
PRISMA flow diagram of the screening process. BIF, borderline intellectual functioning; RCT, randomised controlled trial.^22^

The search identified 4086 records. Of the identified records, 147 were screened on full text, and 119 were excluded as they did not meet the inclusion criteria. In the systematic review, 20 studies were included and 15 of those were included in the meta-analysis. The PRISMA flow diagram is shown in Fig. 1.

### Data extraction

Data extracted from each study included: first author, study title, publication year, country of study, sample size, demographic characteristics, type of intervention (pharmacological, non-pharmacological or combination), type of non-pharmacological intervention (psychosocial, physical activity, diet or occupational therapy), comparator/control details (treatment as usual, waitlist, active comparator or combined), outcome measurement scale (DBS, CBCL, SDQ, ABC, BPI), key results and main conclusions. Missing data were handled by contacting study investigators for unreported data or additional details. All data were extracted by two reviewers working independently, starting on 1 August 2022. Any disagreements were resolved through discussion with a third reviewer.

### Risk of bias

The Cochrane Risk of Bias 2 (RoB 2) tool for randomised trials^28^ was used to assess risk of bias in included studies. This was done by the two reviewers working together, and any disagreements were resolved through discussion with a third reviewer.

The domains assessed using the RoB 2 are: bias arising from the randomisation process, bias due to deviations from intended interventions, bias due to missing outcome data, bias in measurement of the outcome and bias in selection of the reported result. The tool includes a total of 22 items across the 5 domains. A ‘risk-of-bias judgement’ (low risk of bias, some concerns or high risk of bias) is assigned to each domain for every study. Each study is then given an ‘overall risk-of-bias judgement’ based on the risk-of-bias judgement of each of its domains.^28^ We anticipated that there would be a limited number of studies that met our inclusion and exclusion criteria. Therefore, risk-of-bias assessments were not used to exclude studies but to describe the studies included.

### Statistical analysis

Meta-analysis was conducted using Stata version 17 for Windows. Hedges’ *g* was used to estimate the SMD using post-intervention scores obtained between 1 and 18 months post-randomisation.^29^ The SMD was estimated as it is considered more appropriate than a robust variance estimation (RVE) due to the included studies not having multiple comparators with nested effect sizes:

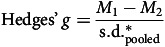

where *M*_1_ is the mean score in group 1, *M*_2_ is the mean score in group 2 and 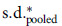 is the pooled and weighted standard deviation.

For studies with more than two intervention groups, we took an average of outcome scores across all intervention groups for the overall meta-analysis. For studies that included more than two comparator groups, we took an average of the outcome scores across all comparator groups. For studies that used several subscales to measure behaviours that challenge, we calculated the Hedges’ *g* for each subscale, and calculated the average of all the Hedges’ *g*. A random-effects meta-analysis estimated the pooled effect size across all 14 studies to determine the overall effect of interventions in reducing levels of behaviours that challenge in children and adolescents with intellectual disabilities:

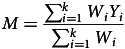

where *Y*_*i*_ is the effect size for each study and *W*_*i*_ is the weight assigned to each study.

Under the random-effects model, the weights account for both within- and between-study variance, whereas under the fixed-effect model, they are solely based on within-study variance. This means that the relative weights assigned to each study are more balanced under the random-effects model compared to the fixed-effect model.^30^

The *I* ^2^ value and prediction interval produced by the random-effects meta-analysis were used to identify the presence of statistical heterogeneity. Other heterogeneity statistics, including *T* ^2^, *H* ^2^ and Cochran’s *Q*, the individual effect size in each study (*θ_i_*) and the true effect size across all studies (*θ*) were also reported. A priori planned subgroup meta-analyses aimed to compare SMDs across three types of interventions (pharmacological, non-pharmacological or combined), across four types of non-pharmacological interventions (psychosocial, physical activity, diet or occupational therapy) and across five measurement scales (BPI, CBCL, SDQ, DBS and ABC). The results of individual studies and syntheses were presented in forest plots.

When studies did not include sufficient raw data to calculate a Hedges’ *g* value, narrative synthesis was used to describe the combined evidence regarding the effectiveness of interventions in reducing levels of behaviours that challenge in children and adolescents with intellectual disabilities. The risk of bias due to missing results was assessed by examining the study characteristics and findings of such studies. Possible small-study effect and publication bias were investigated using a funnel-plot and the Egger regression-based test to test for funnel-plot asymmetry.

## Results

### Study characteristics

Characteristics of the 20 included studies are shown in Supplementary Table S3, and their results are summarised in Table S4. The studies were conducted in the USA, the Netherlands, Italy, Australia, Germany, China, Canada, Denmark, France, New Zealand, Spain, Switzerland, the UK, Belgium, Indonesia, Israel, Sweden and Turkey. Eleven studies investigated non-pharmacological interventions,^31–41^ and nine studies investigated pharmacological interventions.^42–50^ The non-pharmacological interventions include nine psychosocial (Parent–Child Interaction Therapy, movement cognition and emotions, parent training for feeding problems, parent management training, Stepping Stones Triple P (×2), Standing Strong Together, Positive Family Connections intervention, cognitive–behavioural therapy (CBT) targeting emotion regulation) and two diet (Gluten and dairy-containing diet, Ketogenic diet) interventions. The pharmacological interventions include three hormone (growth hormone treatment, intranasal oxytocin, intranasal insulin), two cannabinoid (transdermal cannabidiol gel, cannabidiol), three neurotransmitter modulators (mavoglurant, arbaclofen, levodopa), and one kinase inhibitor (everolimus) interventions. No studies investigated combined pharmacological and non-pharmacological interventions. Nine used the CBCL to measure behaviours that challenge,^31,32,36–38,40,41,48,50^ seven used the ABC,^33,42–46,49^ two used the DBC,^34,47^ one used the SDQ^39^ and one used both the CBCL and ABC.^35^ No studies used the BPI.

A total of 1443 participants were included in the review: of those, 1154 participants were included in the meta-analysis. The mean (s.d.) age of participants was 8.17 (2.27) years. Of the studies that reported the gender of participants (*n* = 1133), 78.52% were male. Furthermore, of the studies that reported the IQ score of participants (*n* = 467), the mean (s.d.) IQ score was 61.68 (26.51).

Six studies included participants with intellectual disabilities or BIF,^32,34,36,37,40,45^ three included participants with global developmental delay,^31,38,41^ eight included participants with specific genetic syndromes (including Fragile X syndrome, Phelan-McDermid syndrome, Prader–Willi syndrome, Angelman syndrome and tuberous sclerosis complex),^42–44,46–50^ one included participants with developmental disabilities (of whom 65% had an intellectual disability, satisfying the inclusion criterion^39^) and two included autistic participants with intellectual disabilities.^33,35^

### Meta-analyses

**Fig. 2:**
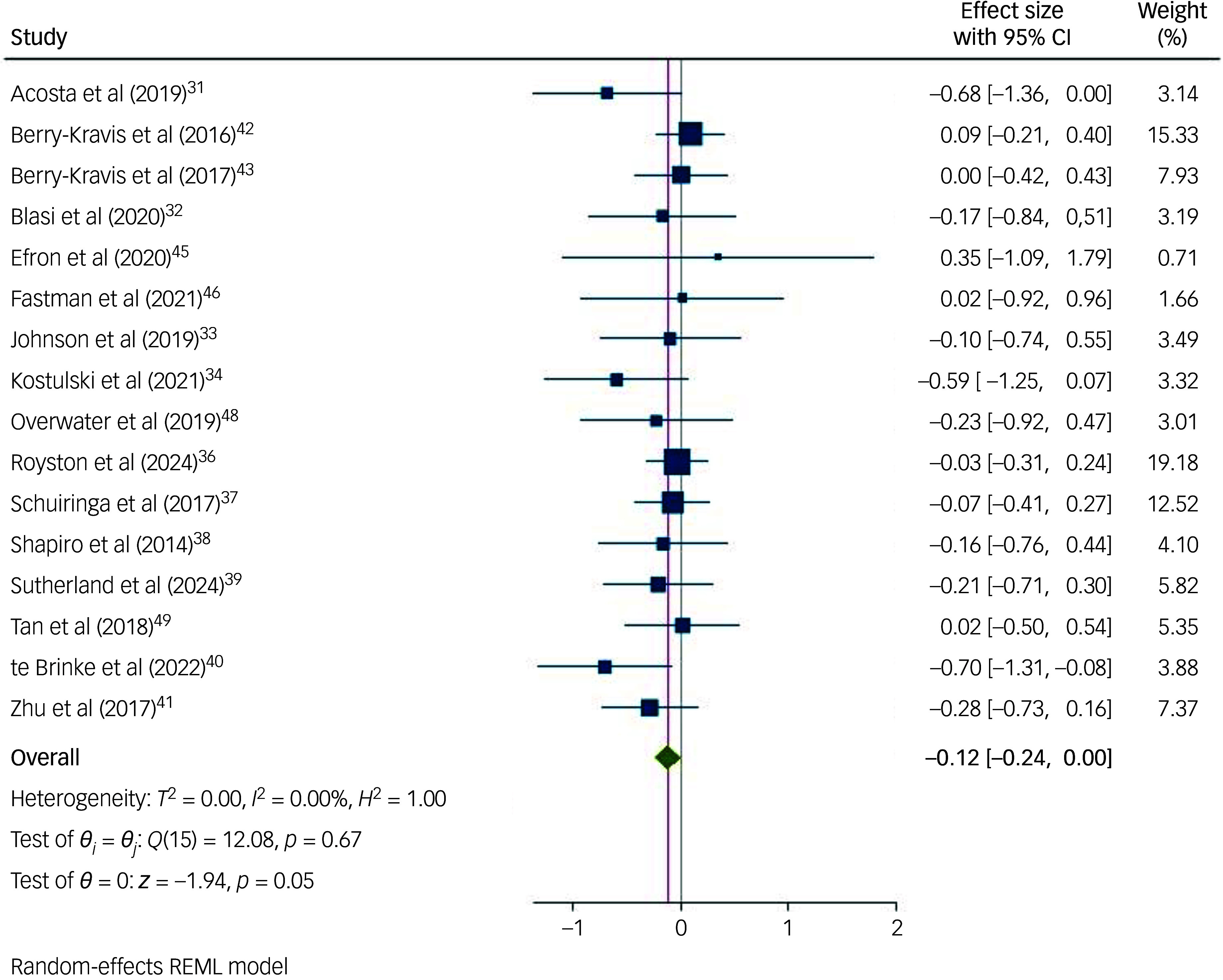
Forest plots of overall random-effect meta-analyses on the effect of all interventions in reducing behaviours that challenge in children with an intellectual disability.

**Fig. 3:**
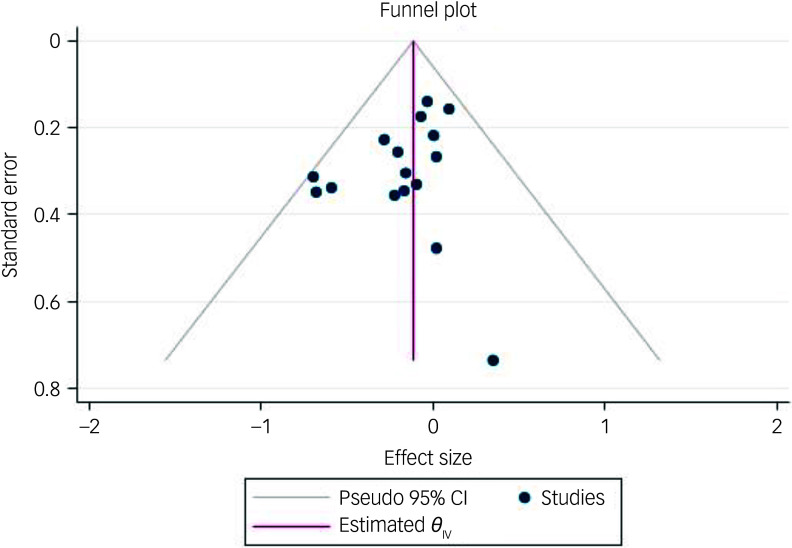
Funnel-plot of the effectiveness of interventions in reducing behaviours that challenge in children with an intellectual disability. REML, Restricted Maximum Likelihood.

Figure 2 presents the individual and combined effect sizes (Hedges’ *g*) of the 16 studies included in the meta-analysis. The random-effects effect size for overall intervention effectiveness was not significant (*n* = 1154; *g* = −0.12; 95% CI [−0.24, 0.00]; *p* = 0.053). There was no evidence of significant heterogeneity in the included studies (*I* ^2^ = 0.00%; 95% prediction interval [−0.25, 0.01]). Furthermore, the funnel-plot (Fig. 3) and Egger’s test found no evidence for small-study effect or publication bias (*z* = −1.46; *p* = 0.146).

### Subgroup meta-analyses

**Fig. 4:**
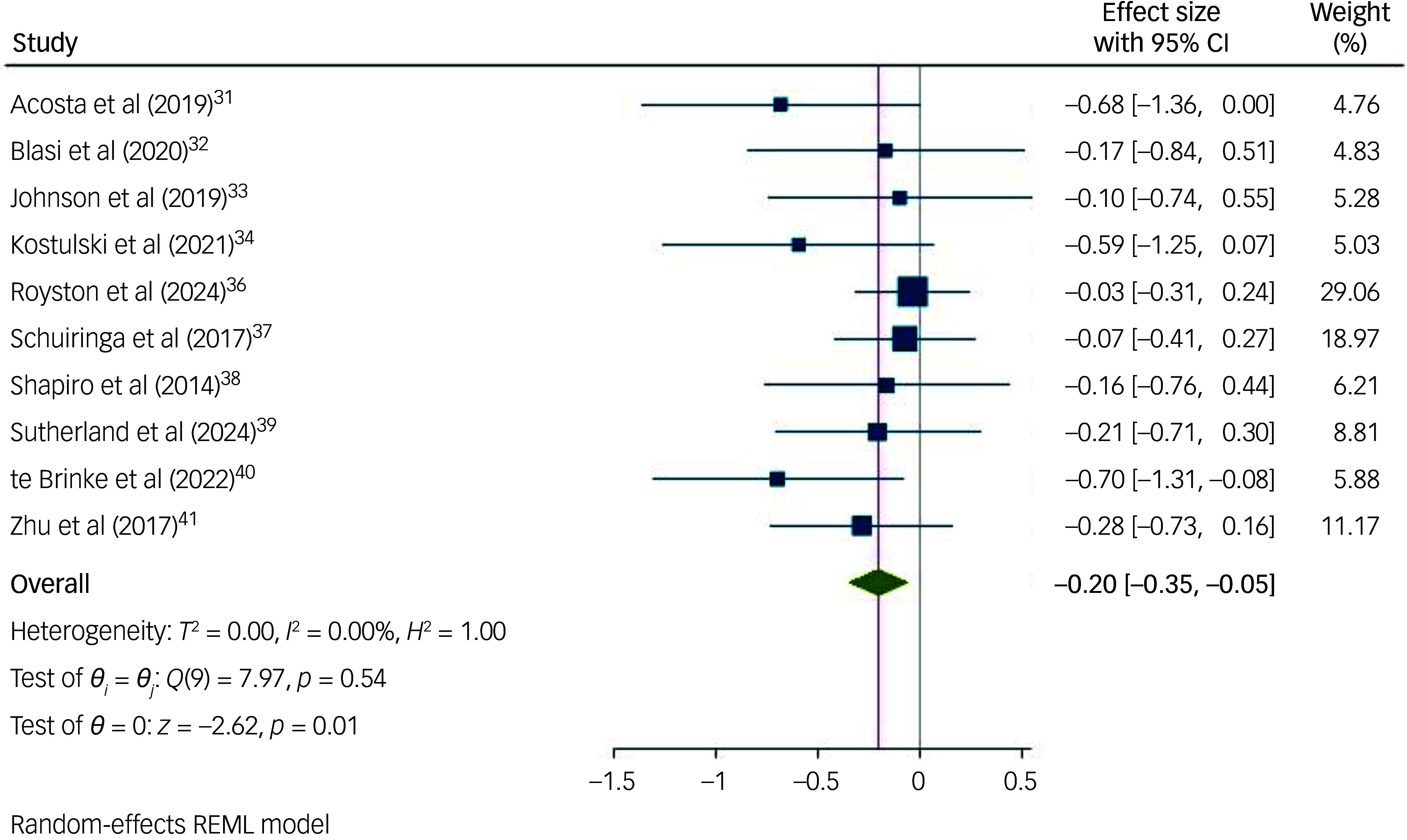
Forest plots of overall random-effect meta-analyses on the effect of non-pharmacological interventions in reducing behaviours that challenge in children with an intellectual disability. REML, Restricted Maximum Likelihood.

**Fig. 5:**
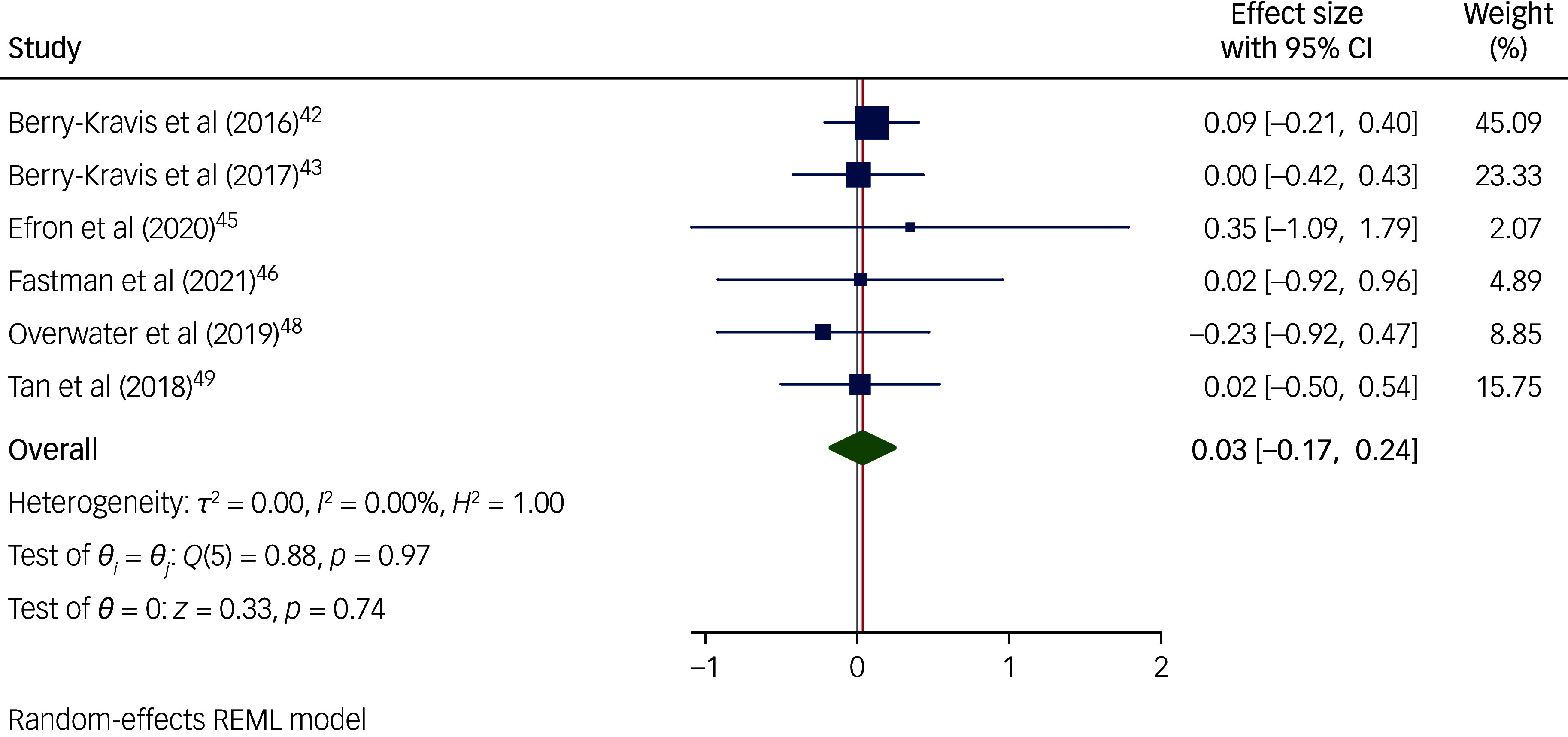
Forest plots of overall random-effect meta-analyses on the effect of pharmacological interventions in reducing behaviours that challenge in children with an intellectual disability. REML, Restricted Maximum Likelihood.

Non-pharmacological interventions (Fig. 4) were significantly effective in reducing behaviours that challenge in children and adolescents with intellectual disabilities (*n* = 747; *g* = −0.20; 95% CI [−0.35, −0.05]; *p* = 0.009; 95% prediction interval [−0.37, −0.02]). Pharmacological interventions (Fig. 5) were not significantly effective in reducing behaviours that challenge in children and adolescents with intellectual disabilities (*n* = 407; *g* = 0.03; 95% CI [−0.17, 0.24]; *p* = 0.742; 95% prediction interval [−0.26, 0.33]). Since no studies investigated combined pharmacological and non-pharmacological interventions, we were unable to examine the effectiveness of such interventions. However, it is important to note that two of the non-pharmacological studies^33,34^ did not exclude participants receiving medication as part of their standard care. Additionally, two of the pharmacological studies^43,45^ did not exclude participants receiving psychosocial support as part of their standard care. The remaining studies did not report information regarding participants’ standard care.

Planned subgroup analyses comparing different types of non-pharmacological interventions were not possible because all but one study^41^ investigated psychosocial interventions.

**Fig. 6:**
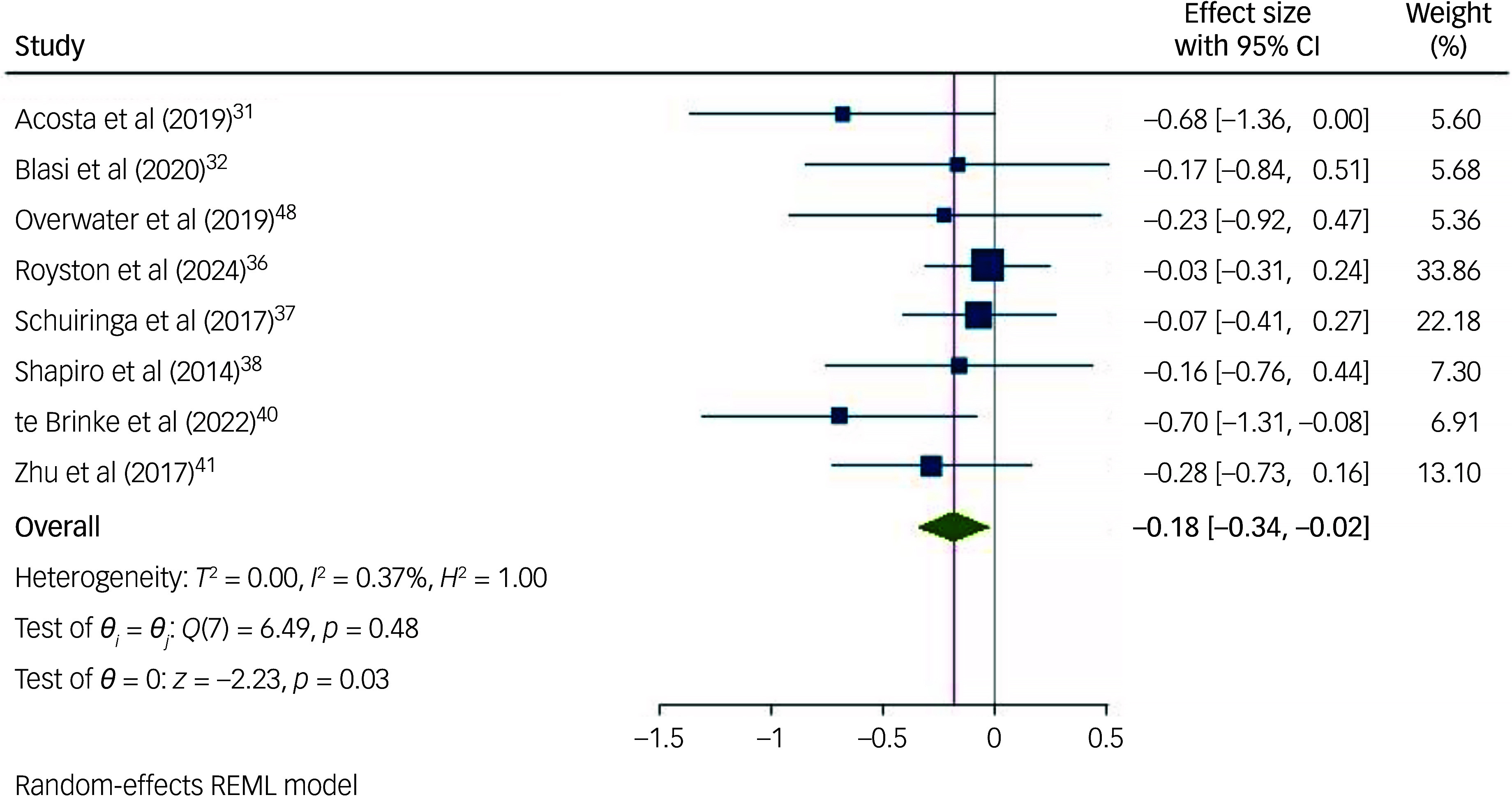
Forest plot of overall random-effect meta-analysis on the effect of interventions in reducing behaviours that challenge in children with an intellectual disability, when using the Child Behaviour Checklist (CBCL) to measure behaviours that challenge. REML, Restricted Maximum Likelihood.

**Fig. 7:**
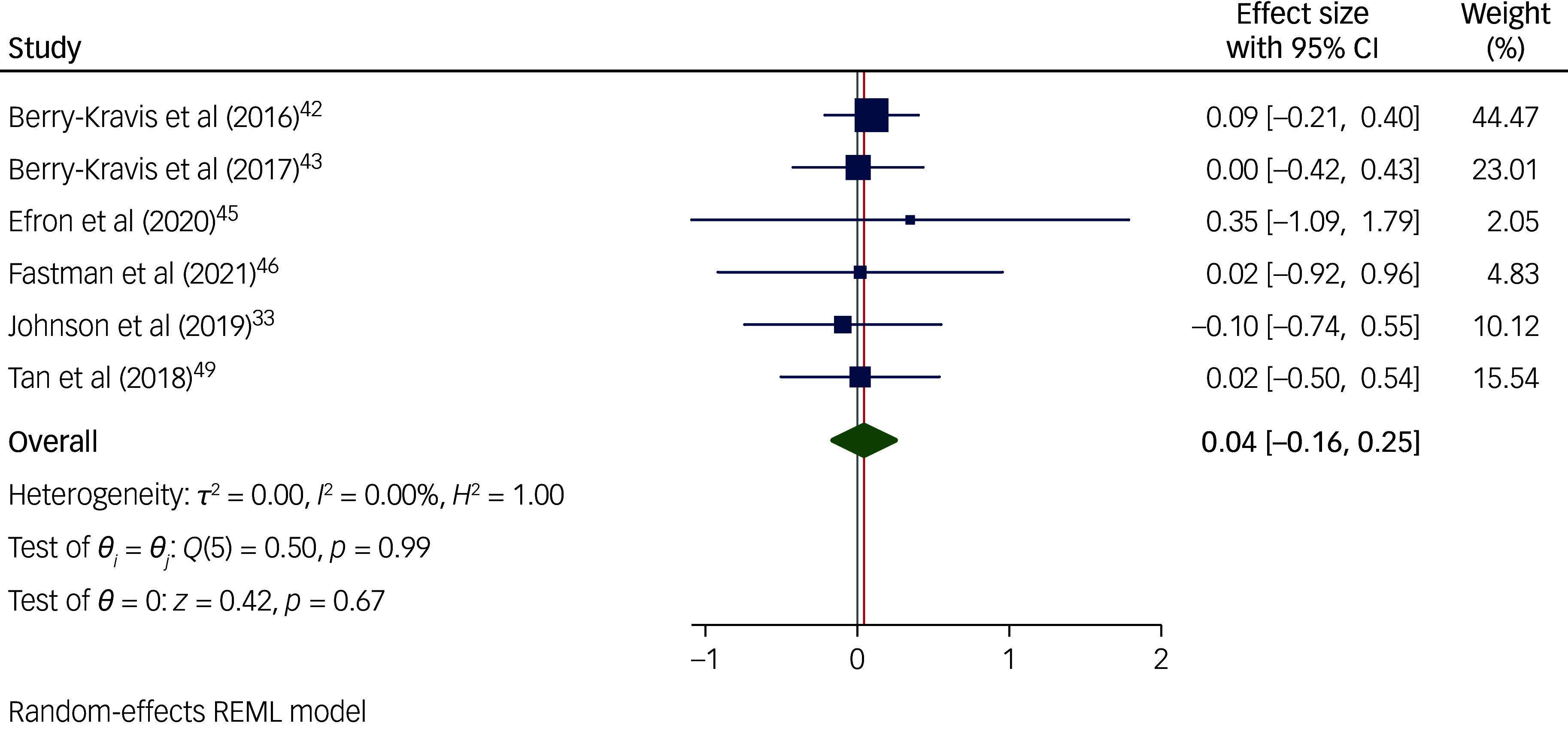
Forest plot of overall random-effect meta-analysis on the effect of interventions in reducing behaviours that challenge in children with an intellectual disability, when using the Aberrant Behaviour Checklist (ABC) to measure behaviours that challenge. REML, Restricted Maximum Likelihood.

Studies using the CBCL to measure behaviours that challenge (Fig. 6) found that interventions were significantly effective in reducing behaviours that challenge in children and adolescents with intellectual disabilities (*n* = 647; *g* = −0.18; 95% CI [−0.34, −0.02]; *p* = 0.026; 95% prediction interval [−0.39, 0.02]). Studies using the ABC (Fig. 7) were associated with a non-significant effect size (*n* = 412; *g* = 0.04; 95% CI [−0.16, 0.25]; *p* = 0.673; 95% prediction interval [−0.247, 0.336]). Meta-analyses for studies using the other measures were not possible because only two studies used the DBC, one study used the SDQ and no studies used the BPI.

### Risk of bias due to missing results

Four studies were included in this review but excluded from the meta-analysis due to not reporting sufficient data on behaviours that challenge to allow the extraction of a study effect size.^35,44,47,50^ These studies investigated cannabidiol (pharmacological), growth hormone treatment (pharmacological), gluten and dairy-containing diets (non-pharmacological) and intranasal insulin (pharmacological), respectively.

All four studies reported no significant effects of interventions in reducing behaviours that challenge in children and adolescents with intellectual disabilities. They were all reasonably small trials, collectively making up 20.03% (*n* = 289/1443) of total participants included in this review. We concluded that the inability to include results from these studies in the meta-analysis was unlikely to bias or change the overall non-significant effect size. We also concluded that the inability to include these results in their respective intervention-type subgroup analyses (pharmacological/non-pharmacological) was unlikely to bias or change their effect sizes.

### Risk of bias

Risk-of-bias scores for each included study are shown in Table 2. Overall, 3 studies were evaluated as having ‘low risk of bias’ and 17 studies as having ‘some concerns’.

**Table 2:** Risk-of-bias assessment of the included studies assessed using the Cochrane Risk of Bias 2 (RoB 2) tool^28^

Study	Randomisation process	Deviations from intended interventions	Missing outcome data	Measurement of the outcome	Selection of the reported result	Overall risk of bias
Acosta et al (2019)^31^	Low	Some concerns	Low	Some concerns	Low	Some concerns
Berry-Kravis et al (2016)^42^	Low	Low	Low	Low	Some concerns	Some concerns
Berry-Kravis et al (2017)^43^	Low	Low	Low	Low	Some concerns	Some concerns
Berry-Kravis et al (2022)^44^	Low	Low	Low	Low	Low	Low
Blasi et al (2020)^32^	Low	Some concerns	Low	Low	Some concerns	Some concerns
Efron et al (2020)^45^	Low	Low	Low	Low	Some concerns	Some concerns
Fastman et al (2021)^46^	Some concerns	Low	Low	Low	Low	Some concerns
Johnson et al (2019)^33^	Low	Some concerns	Low	Some concerns	Low	Some concerns
Kostulski et al (2021)^34^	Some concerns	Some concerns	Low	Some concerns	Some concerns	Some concerns
Lo et al (2015)^47^	Low	Low	Low	Some concerns	Some concerns	Some concerns
Navarro et al (2015)^35^	Low	Low	Low	Low	Low	Low
Overwater et al (2019)^48^	Low	Low	Low	Low	Some concerns	Some concerns
Royston et al (2024)^36^	Low	Low	Low	Low	Low	Low
Schuiringa et al (2017)^37^	Low	Low	Some concerns	Some concerns	Low	Some concerns
Shapiro et al (2014)^38^	Low	Low	Low	Some concerns	Low	Some concerns
Sutherland et al (2024)^39^	Some concerns	Low	Low	Some concerns	Low	Some concerns
Tan et al (2018)^49^	Low	Some concerns	Low	Low	Some concerns	Some concerns
te Brinke et al (2022)^40^	Low	Low	Low	Some concerns	Low	Some concerns
Zhu et al (2017)^41^	Low	Some concerns	Low	Low	Low	Some concerns
Zwanenburg et al (2016)^50^	Low	Low	Low	Low	Some concerns	Some concerns

## Discussion

This review aimed to synthesise recent evidence on the effectiveness of interventions to reduce behaviours that challenge in children with intellectual disabilities by investigating all types of interventions published after November 2014. The meta-analysis indicated a small and non-significant effect size for overall intervention effectiveness suggesting current interventions were, overall, not effective in reducing behaviours that challenge in our target population. The prediction interval suggests that future studies are likely to replicate this finding.

Subgroup meta-analyses indicated that non-pharmacological interventions showed significant and consistent benefit in reducing behaviours that challenge in children and adolescents with intellectual disabilities. The prediction interval suggests that future studies are likely to replicate this benefit. This finding is consistent with the NICE^12^ reviews on the effectiveness of non-pharmacological interventions (environmental changes and psychosocial) in all age groups. It is worth noting that the NICE^12^ reviews were mainly based on studies with individuals with autism, where a high proportion of participants had intellectual disabilities. This is also consistent with findings from previous reviews on adults^9,51^ and mixed-aged participants,^15,18^ which suggested that non-pharmacological interventions were effective in reducing behaviours that challenge in all age groups. Non-pharmacological interventions in the present review included studies investigating mostly psychosocial (behavioural therapies, CBT, parent–child interaction therapies and parent training programmes) and diet interventions (two studies only). Previous reviews included studies investigating different types of non-pharmacological interventions. Bruinsma et al^9^ investigated psychosocial (CBT, multisensory therapy and mindfulness therapy) and behavioural interventions. Chowdhury and Benson^51^ and Denis et al^15^ investigated behavioural (differential reinforcement and non-intrusive forms of reinforcement) interventions while Ogg-Groenendaal et al^18^ investigated lifestyle interventions. All the evidence taken together, suggests that a range of non-pharmacological interventions may be effective in reducing behaviours that challenge, though most evidence is available for psychosocial interventions.

The meta-analysis indicated no significant effects of pharmacological interventions in reducing behaviours that challenge in children and adolescents with intellectual disabilities. The prediction interval suggests uncertainty regarding the effects of those treatments within the context of the included studies in this review. This highlights the challenges in designing and conducting new RCTs testing pharmacological agents without fully understanding the underlying cause of heterogeneity across the studies. Other methodologies, such as use of routine data, if available, can also be used to corroborate, or otherwise, the impact of pharmacological interventions on behaviours that challenge and therefore, support better clinical decision-making. Previous reviews on the effectiveness of pharmacological interventions in reducing behaviours that challenge have found mixed evidence. Meta-analyses specific to children and adolescents with intellectual disabilities have shown that some pharmacological interventions (antipsychotics) are effective in reducing behaviours that challenge, while others have inconclusive evidence (anticonvulsants and antioxidants).^8,12^ However, findings from this review are not directly comparable because previous reviews did not investigate the overall effectiveness of all types of pharmacological interventions, and we did not explore the effectiveness of each type of pharmacological intervention. The studies included in our review investigated the following medications and pharmaceutical or chemical agents: hormones (insulin, growth hormone and oxytocin), cannabinoids (cannabidiol), receptor antagonists (levodopa, arbaclofen, mavoglurant) and kinase inhibitors (everolimus). These differ from the pharmacological interventions included in previous reviews (antipsychotics, antioxidants, anticonvulsants and biomedical interventions), and this may account for the difference in findings. The lack of efficacy of pharmacological interventions in the present review may also be associated with the design of the included studies. Except for the Berry-Kravis studies,^42–44^ the pharmacological studies included in this review included small sample sizes (*n* = 7−55). Crucially, four out of the nine pharmacological studies did not conduct a priori power analyses.^45–47,50^

The results indicated significant reduction in behaviours that challenge when measured using the CBCL but not the ABC. However, prediction intervals suggest that future studies using either the CBCL or ABC might not replicate the observed effects. These results caution against drawing firm conclusions of effectiveness when using either scale. The difference in findings might be because the ABC was developed for adults with intellectual disabilities,^24^ whereas the CBCL was developed for children.^7^ In a review by Aman et al,^52^ the ABC detected a smaller reduction in behaviour problems among young people with developmental disabilities following intervention than other scales used (e.g. various versions of Conners’ Rating Scale, and the Swanson, Nolan and Pelham SNAP Rating Scale). It is likely that the CBCL is the most sensitive to change measure of behaviours that challenge in children with intellectual disabilities. In Buckley et al’s meta-analysis,^4^ the CBCL detected the highest prevalence rates of behaviours that challenge in children with intellectual disabilities even compared to the DBC, which was developed specifically for individuals with intellectual disabilities. It is worth highlighting that the CBCL was developed as a wide-range assessment of emotional and behavioural difficulties in children, whereas the ABC was developed to measure behaviours that challenge specifically.^7,24^ This means that in addition to including items clearly relevant to the concept of behaviours that challenge, the CBCL also assesses other aspects of children’s behaviour, which may be of concern, but would not generally be regarded as behaviours that challenge.

Another reason for the difference in findings between the CBCL and ABC effect sizes could be that most of the included studies using the CBCL investigated non-pharmacological interventions (*N* = 7/9), whereas most of the studies using the ABC investigated pharmacological interventions (*N* = 5/7). Given our subgroup meta-analysis showed that non-pharmacological interventions were significantly effective in reducing behaviours that challenge but pharmacological interventions were not, measurement scale findings might reflect a difference in intervention effectiveness rather than scale sensitivity.

Finally, it has been reported that a common issue with the analysis of scores derived from measures of behaviours that challenge is skewness.^53^ The studies included in the present review did not report whether skewness was observed and all studies included scores measured in the original scale of the measure. Therefore, we were unable to assess whether skewness might have added bias to the effect-size estimation.

### Strengths and weaknesses

To the best of our knowledge, this is the first meta-analysis to investigate the effectiveness of all types of interventions in reducing behaviours that challenge specifically in children with intellectual disabilities. Our review included RCTs and operationalised behaviours that challenge by using well-validated and standardised scales. Additionally, the funnel-plot and Egger’s test found no evidence of publication bias caused by the studies included. Although we searched for studies of all languages and countries, most studies included were conducted in Western countries. This means that findings might not be generalisable to non-Western countries. Another limitation is that the majority of the included RCTs had small sample sizes and did not conduct a priori power analyses. Considering this with the fact that most studies had a risk-of-bias rating of ‘some concerns’, this likely undermines the certainty of the findings. As in previous reviews,^8^ longer-term effectiveness could not be established because included studies mostly did not include a follow-up point beyond the immediate post-intervention assessment (except for Lo,^47^ Shapiro^38^ and Zhu^41^). Despite contacting the authors, we were not able to retrieve the full text of nine papers that passed stage one of our screening process. Therefore, we were unable to determine the eligibility of such studies at full-text stage and relevant findings might have been missed out. Finally, another limitation is that we grouped together different types of interventions to calculate effect sizes. The intention was to replicate the approach taken by the NICE^12^ review, by grouping interventions based on their mechanisms of change. While this approach allowed for an overview of the effectiveness of pharmacological and non-pharmacological interventions, it might have obscured important differences in effectiveness between different intervention types within the two groups. Planned meta-analyses of interventions grouped by more specific mechanisms of change were not possible due to the low number of studies identified in each category.

### Implications for practice, policy and future research

Present findings support the recommendation by NICE^12^ that non-pharmacological interventions should be first-line treatment for children and adolescents with intellectual disabilities and behaviours that challenge. While the present review could not compare different types of non-pharmacological interventions, all but two included studies focused on psychosocial approaches, mostly parent training programmes, CBT and others. There is a need for more targeted investigations into the effectiveness of psychological therapies for children and adolescents with intellectual disabilities,^54^ and the present review found that the body of available evidence is mostly about parent training.

Overall, this systematic review and meta-analysis is the first since the NICE^12^ review to focus on children with intellectual disabilities and behaviours that challenge, using only RCTs. It indicates that the pharmacological interventions evaluated in research published since 2014 (including mostly hormones, cannabinoids and receptor antagonists) are not effective in reducing behaviours that challenge amongst children with intellectual disabilities, whereas non-pharmacological approaches (mostly psychosocial) are. Pharmacological interventions were associated with a non-significant and near zero effect size, whereas non-pharmacological interventions were associated with a small, albeit significant, effect size (*g* = −0.20; 95% CI [−0.35, −0.05]; *p* = 0.009), indicating a reduction in behaviours that challenge. This was particularly the case for studies that measured behaviours that challenge using the CBCL. Most of these studies measured the CBCL total score, which includes both internalising and externalising outcomes. Findings were limited to post-intervention effectiveness while longer-term effects were mostly not measured in the included studies. Overall, the findings suggest that we need to increase the effort in identifying effective treatments for behaviours that challenge in this population as currently researched approaches are likely not effective, except for psychosocial interventions. Future research must embrace greater specificity by looking at topography, rather than behaviours that challenge as an overall construct, and should focus on the fidelity of behaviours that challenge measurement scales. Future work should include more rigorously conducted RCTs to evaluate pharmacological interventions based on clear mechanistic insights, while also considering alternative methodologies, such as utilising routine clinical data and non-pharmacological approaches, to supplement this.

## Supporting information

Lye et al. supplementary material Lye et al. supplementary material

## Data Availability

The data that support the findings of this study are available from the corresponding author, V.L., upon reasonable request.

## Supplementary material

The supplementary material is available online at https://doi.org/10.1192/bjo.2025.10871
