# Supplementary material for: Behaviours that challenge in children with intellectual disability: systematic review and meta-analysis of pharmacological and non-pharmacological interventions
Source: BJPsych Open. 2025 Oct 27;11(6):e256. doi: 10.1192/bjo.2025.10871 (PMC12569614; doi:10.1192/bjo.2025.10871)
Supplement: Lye et al. supplementary material [file S2056472425108715sup001.docx]

**Table S1.** *PRISMA 2020 Checklist (Page et al., 2020).*

| **Section and Topic** | **Item #** | **Checklist item** | **Location where item is reported** |
| --- | --- | --- | --- |
| **TITLE** | | |  |
| Title | 1 | Identify the report as a systematic review. | p.1 & p.3 |
| **ABSTRACT** | | |  |
| Abstract | 2 | See the PRISMA 2020 for Abstracts checklist. | p.2 |
| **INTRODUCTION** | | |  |
| Rationale | 3 | Describe the rationale for the review in the context of existing knowledge. | p.3 – p.6 |
| Objectives | 4 | Provide an explicit statement of the objective(s) or question(s) the review addresses. | p.5 – p.6 |
| **METHODS** | | |  |
| Eligibility criteria | 5 | Specify the inclusion and exclusion criteria for the review and how studies were grouped for the syntheses. | p.7 – p.8 |
| Information sources | 6 | Specify all databases, registers, websites, organisations, reference lists and other sources searched or consulted to identify studies. Specify the date when each source was last searched or consulted. | p.6 – p.7 |
| Search strategy | 7 | Present the full search strategies for all databases, registers and websites, including any filters and limits used. | p.6 & Supplementary Table S2 |
| Selection process | 8 | Specify the methods used to decide whether a study met the inclusion criteria of the review, including how many reviewers screened each record and each report retrieved, whether they worked independently, and if applicable, details of automation tools used in the process. | p.7 – p.8 |
| Data collection process | 9 | Specify the methods used to collect data from reports, including how many reviewers collected data from each report, whether they worked independently, any processes for obtaining or confirming data from study investigators, and if applicable, details of automation tools used in the process. | p.8 – p.9 |
| Data items | 10a | List and define all outcomes for which data were sought. Specify whether all results that were compatible with each outcome domain in each study were sought (e.g. for all measures, time points, analyses), and if not, the methods used to decide which results to collect. | p.8 |
|  | 10b | List and define all other variables for which data were sought (e.g. participant and intervention characteristics, funding sources). Describe any assumptions made about any missing or unclear information. | p.8 |
| Study risk of bias assessment | 11 | Specify the methods used to assess risk of bias in the included studies, including details of the tool(s) used, how many reviewers assessed each study and whether they worked independently, and if applicable, details of automation tools used in the process. | p.9 |
| Effect measures | 12 | Specify for each outcome the effect measure(s) (e.g. risk ratio, mean difference) used in the synthesis or presentation of results. | p.9 |
| Synthesis methods | 13a | Describe the processes used to decide which studies were eligible for each synthesis (e.g. tabulating the study intervention characteristics and comparing against the planned groups for each synthesis (item #5)). | p.9 – p.10 |
|  | 13b | Describe any methods required to prepare the data for presentation or synthesis, such as handling of missing summary statistics, or data conversions. | p.9 – p.10 |
|  | 13c | Describe any methods used to tabulate or visually display results of individual studies and syntheses. | p.9 – p.10 |
|  | 13d | Describe any methods used to synthesize results and provide a rationale for the choice(s). If meta-analysis was performed, describe the model(s), method(s) to identify the presence and extent of statistical heterogeneity, and software package(s) used. | p.9 – p.10 |
|  | 13e | Describe any methods used to explore possible causes of heterogeneity among study results (e.g. subgroup analysis, meta-regression). | p.9 – p.10 |
|  | 13f | Describe any sensitivity analyses conducted to assess robustness of the synthesized results. | p.9 – p.10 |
| Reporting bias assessment | 14 | Describe any methods used to assess risk of bias due to missing results in a synthesis (arising from reporting biases). | p.9 – p.10 |
| Certainty assessment | 15 | Describe any methods used to assess certainty (or confidence) in the body of evidence for an outcome. | p.9 – p.10 |
| **RESULTS** | | |  |
| Study selection | 16a | Describe the results of the search and selection process, from the number of records identified in the search to the number of studies included in the review, ideally using a flow diagram. | p.8 & Figure 1 |
|  | 16b | Cite studies that might appear to meet the inclusion criteria, but which were excluded, and explain why they were excluded. | Figure 1 |
| Study characteristics | 17 | Cite each included study and present its characteristics. | Supplementary Table S3 |
| Risk of bias in studies | 18 | Present assessments of risk of bias for each included study. | p.13 & Table 2 |
| Results of individual studies | 19 | For all outcomes, present, for each study: (a) summary statistics for each group (where appropriate) and (b) an effect estimate and its precision (e.g. confidence/credible interval), ideally using structured tables or plots. | Figure 2 & p.13 |
| Results of syntheses | 20a | For each synthesis, briefly summarise the characteristics and risk of bias among contributing studies. | p.11 & p.13 |
|  | 20b | Present results of all statistical syntheses conducted. If meta-analysis was done, present for each the summary estimate and its precision (e.g. confidence/credible interval) and measures of statistical heterogeneity. If comparing groups, describe the direction of the effect. | Figures 2, 4–7 |
|  | 20c | Present results of all investigations of possible causes of heterogeneity among study results. | p.12 & Figure 3 |
|  | 20d | Present results of all sensitivity analyses conducted to assess the robustness of the synthesized results. | p.12 & Figure 3 |
| Reporting biases | 21 | Present assessments of risk of bias due to missing results (arising from reporting biases) for each synthesis assessed. | p.13 |
| Certainty of evidence | 22 | Present assessments of certainty (or confidence) in the body of evidence for each outcome assessed. | Figures 2, 4–7 |
| **DISCUSSION** | | |  |
| Discussion | 23a | Provide a general interpretation of the results in the context of other evidence. | p.14 – p.16 |
|  | 23b | Discuss any limitations of the evidence included in the review. | p.16 – p.17 |
|  | 23c | Discuss any limitations of the review processes used. | p.16 – p. 17 |
|  | 23d | Discuss implications of the results for practice, policy, and future research. | p.17 |
| **OTHER INFORMATION** | | |  |
| Registration and protocol | 24a | Provide registration information for the review, including register name and registration number, or state that the review was not registered. | p.6 |
|  | 24b | Indicate where the review protocol can be accessed, or state that a protocol was not prepared. | p.6 |
|  | 24c | Describe and explain any amendments to information provided at registration or in the protocol. | n/a |
| Support | 25 | Describe sources of financial or non-financial support for the review, and the role of the funders or sponsors in the review. | p.19 |
| Competing interests | 26 | Declare any competing interests of review authors. | p.19 |
| Availability of data, code and other materials | 27 | Report which of the following are publicly available and where they can be found: template data collection forms; data extracted from included studies; data used for all analyses; analytic code; any other materials used in the review. | p.19 – p.20 |

*From:*  Page MJ, McKenzie JE, Bossuyt PM, Boutron I, Hoffmann TC, Mulrow CD, et al. The PRISMA 2020 statement: an updated guideline for reporting systematic reviews. BMJ 2021;372:n71. doi: 10.1136/bmj.n71

**Table S2.** *Full search strategies for the following electronic biographical databases: PsychINFO, MEDLINE, EMBASE and Web of Science.*

| Database(s) | Full Search Strategy |
| --- | --- |
| PsychINFO, MEDLINE and EMBASE Ovid | 1 (learning disab* or learning difficult* or learning impair* or intellectual* disab* or intellectual* impair* or borderline intellectual* or development* disab* or development* disorder* or developmental delay or development* impair* or intellectual developmental disorder or mental* deficien* or mental* retard* or mental* handicap* or mental* disab* or mental* impair* or mental* challenged or mental* subnormal* or mental* sub-normal* or subaverage intelligence or subnormal or cognitive delay).mp. [mp=title, abstract, original title, name of substance word, subject heading word, floating sub-heading word, keyword heading word, organism supplementary concept word, protocol supplementary concept word, rare disease supplementary concept word, unique identifier, synonyms]  2 (Developmental Behavi* Checklist* or DBC* or Aberrant Behavi* Checklist* or ABC* or Behavi* Problem* Inventory or BPI* or BPI-S or Child Behavi* Checklist* or CBCL* or "Strength* and Difficult* Questionnaire*" or SDQ* or challenging behavi* or maladaptive behavi* or aberrant behavi* or problem behavi* or behavi* problem* or behav* challenging or behav* disrupt* or behav* disturb* or behav* problem* or aggress* or destructive behavi* or property destruction or disruptive behavi* or stereotyp* or stereotyped behavi* or Repetitive behave* or self-injur* or self-injurious behavi* or SIB or self-harm or self hurt* or self wound* or ruminat* or Self damage* or self destruct* or self mutilat* or self violen* or parasuicid* or para-suicid*).mp. [mp=title, abstract, original title, name of substance word, subject heading word, floating sub-heading word, keyword heading word, organism supplementary concept word, protocol supplementary concept word, rare disease supplementary concept word, unique identifier, synonyms]  3 (randomi* controlled trial* or RCT* or Clinical trial* or crossover or "cross over" or single* blind* or doubl* blind* or tripl* blind* or singleblind* or doubleblind* or tripleblind* or placebo* or random*).mp. [mp=title, abstract, original title, name of substance word, subject heading word, floating sub-heading word, keyword heading word, organism supplementary concept word, protocol supplementary concept word, rare disease supplementary concept word, unique identifier, synonyms]  4 1 and 2 and 3  5 limit 4 to (humans and yr="2014 -Current") |
| Web of Science | learning disab* or learning difficult* or learning impair* or intellectual* disab* or intellectual* impair* or borderline intellectual* or development* disab* or development* disorder* or developmental delay or development* impair* or intellectual developmental disorder or mental* deficien* or mental* retard* or mental* handicap* or mental* disab* or mental* impair* or mental* challenged or mental* subnormal* or mental* sub-normal* or suvaverage intelligence or subnormal or cognitive delay (Topic) and Developmental Behavi* Checklist* or DBC* or Aberrant Behavi* Checklist* or ABC* or Behavi* Problem* Inventory or BPI* or BPI-S or Child Behavi* Checklist* or CBCL* or "Strength* and Difficult* Questionnaire*" or SDQ* or challenging behavi* or maladaptive behavi* or aberrant behavi* or problem behavi* or behavi* problem* or behav* challenging or behav* disrupt* or behav* disturb* or behav* problem* or aggress* or destructive behavi* or property destruction or disruptive behavi* or stereotyp* or stereotyped behavi* or Repetitive behave* or self-injur* or self-injurious behavi* or SIB or self-harm or self hurt* or self wound* or ruminat* or Self damage* or self destruct* or self mutilat* or self violen* or parasuicid* or para-suicid* (Topic) and randomi* controlled trial* or RCT* or Clinical trial* or crossover or "cross over" or single* blind* or doubl* blind* or tripl* blind* or singleblind* or doubleblind* or tripleblind* or placebo* or random* (Topic) and child* OR adolescen* OR boy* OR girl* OR youth OR teenag* OR young OR student* OR youngster* (Topic) |

| **Table S3.** *Characteristics of the Included Randomised Controlled Trials (n=20)* | | | | | | | |
| --- | --- | --- | --- | --- | --- | --- | --- |
| **Study** | **Study Location** | **Groups** | **Participant Characteristics** | **Type of Intervention** | **Comparator** | **Primary Outcome of the Study** | **Behaviours that Challenge Measure** |
| Acosta et al. (2019)^2^ | United States | IG (n=15)  CG (n=19) | Population: Children with Developmental Delay (DD) or Borderline DD;  % with ID, global DD, or BIF: 100%;  Mean (SD) age: 49.19 (13.10) months;  Sex: 76.00% male;  Mean (SD) IQ score: 62.20 (11.70) | Non-Pharmacological (Psychosocial):  Parent-Child Interaction Therapy | Waitlist | Both sleep and behaviour problems | CBCL subscale: Sleep Problems |
| Berry-Kravis et al. (2016)^7^ | 16 countries, including:  Australia, Canada, Denmark, France, Germany, Italy, Spain, Switzerland, UK, United States, Belgium, Indonesia, Israel, Netherlands, Sweden, and Turkey | IG-1 (n=31)  IG-2 (n=27)  IG-3 (n=39)  CG (n=41) | Population: Adolescents with fragile X syndrome;  % with ID, global DD, or BIF: 100%; Mean (SD) age: 14.50 (1.74) years;  Mean (SD) IQ score: 40.00 (44.00) | Pharmacological:  Mavoglurant | Placebo | Behavioural symptoms | ABC-Community Edition using the FXS-specific algorithm (CFX) subscales: Irritability, Lethargy/ withdrawal, Stereotypic behavior, Hyperactivity, Inappropriate speech, Social avoidance |
| Berry-Kravis et al. (2017)^8^ | United States | IG-1 (n=38)  IG-2 (n=39)  IG-3 (n=38)  CG (n=44) | Population: Children with fragile X syndrome;  % with ID, global DD, or BIF: 100%; Mean (SD) age: 7.78 (1.99) years;  Sex: 83.70% male | Pharmacological: Arbaclofen | Placebo | Social avoidance | ABC-CFX subscales:  Social Avoidance, Irritability, Hyperactivity, Stereotypic behavior, Lethargy, Inappropriate speech |
| Berry-Kravis et al. (2022)^9^ | United States, Australia and New Zealand | IG (n=109)  CG (n=101) | Population: Children and adolescents with a diagnosis of Fragile X syndrome and body mass index of 12–30 kg/m2;  % with ID, global DD, or BIF: 100%;  Mean age: 9.70 years;  Sex: 75.00% male | Pharmacological: Transdermal Cannabidiol Gel | Placebo | Social avoidance | Aberrant Behavior Checklist–Community Edition FXS (ABC-CFXS) subscales:  Social Avoidance, Irritability, Social Unresponsiveness/ Lethargy |

| **Table S3.** *(Continued.)* | | | | | | | |
| --- | --- | --- | --- | --- | --- | --- | --- |
| **Study** | **Study Location** | **Groups** | **Participant Characteristics** | **Type of Intervention** | **Comparator** | **Primary Outcome** | **Behaviours that Challenge Measure** |
| Blasi et al. (2020)^10^ | Italy | IG (n=16)  CG (n=16) | Population: Children with Borderline Intellectual Functioning;  % with ID, global DD, or BIF: 100%; Mean (SD) age: 8.23 (1.36) years;  Mean (SD) IQ score: 76.86 (7.87) | Non-Pharmacological (Psychosocial): The movement cognition and narration of the emotions (MCNT) | Treatment as Usual:  Standard speech therapy (SST) | Behaviour problems | CBCL total |
| Efron et al. (2020)^17^ | Australia | IG (n=3)  CG (n=4) | Population: Children and adolescents with intellectual disability;  % with ID, global DD, or BIF: 100%; Mean (range) age: 14.50 (11.00–16.90) years;  Sex: 64.00% male | Pharmacological: Cannabidiol | Placebo | Severe behavioural problems | ABC subscales: Irritability, Social withdrawal, Stereotypic behaviour, Hyperactivity/non-compliance, Inappropriate speech |
| Fastman et al. (2021)^21^ | United States | IG (n=7)  CG (n=9) | Population: Children and Adolescents with Phelan-McDermid syndrome;  % with ID, global DD, or BIF: 100%; Mean (SD, range) age: 8.40 (3.20, 5–17) years;  Sex: 50.00% male | Pharmacological:  Intranasal oxytocin | Placebo | Social withdrawal | ABC subscales: Irritability, Social withdrawal, Stereotypy, Hyperactivity, Inappropriate speech |
| Johnson et al. (2019)^26^ | United States | IG (n=17) CG (n=20) | Population: Children with Autism Spectrum Disorder;  % with ID, global DD, or BIF: 52.4%;  Mean (SD) age: 5.10 (1.40) years;  Sex: 95.60% male | Non-Pharmacological (Psychosocial): Parent Training for Feeding Problems | Waitlist | Child feeding outcomes | ABC subscale:  Irritability (agitation, aggression, and self-injurious behaviors) |
| Kostulski et al. (2021)^27^ | Germany | IG (n=17) CG (n=18) | Population: Children with intellectual disability;  % with ID, global DD, or BIF: 100%; Mean (SD, range) age: 11.19 (2.87, 6–16) years;  Sex: 86.00% male | Non-Pharmacological (Psychosocial): Parent management training (PMT) | Waitlist | Behavioural and emotional problems | German version of the DBC: the VFE |

| **Table S3.** *(Continued.)* | | | | | | | |
| --- | --- | --- | --- | --- | --- | --- | --- |
| **Study** | **Study Location** | **Groups** | **Participant Characteristics** | **Type of Intervention** | **Comparator** | **Primary Outcome** | **Behaviours that Challenge Measure** |
| Lo et al. (2015)^28^ | The Netherlands | IG (n=24) CG (n=18) | Population: Children with Prader–Willi syndrome;  % with ID, global DD, or BIF: 100%; Median (IQR) age: 6.40 (4.90, 7.60) years;  Sex: 45.00% male;  Median (IQR) IQ score: 65.00 (59.00, 83.00) | Pharmacological: Growth Hormone Treatment | No treatment | Social behavioural problems | DBC total |
| Navarro et al. (2015)^33^ | United States | IG (n=6)  CG (n=6) | Population: Children with Autism Spectrum Disorders;  % with ID, global DD, or BIF: 100%; Median (IQR) age: 5.80 (5.00–7.00) years;  Median (IQR) IQ score: 58.30 (48.00–76.00) | Non-Pharmacological (Diet): Gluten and dairy containing diet | Placebo | Intestinal permeability (IP) and behaviour | ABC subscales: Hyperactivity, Irritability; and CBCL subscale: Inattention |
| Overwater et al. (2019)^36^ | The Netherlands | IG (n=15) CG (n=17) | Population: Children and adolescents with Tuberous Sclerosis Complex;  % with ID, global DD, or BIF: 100%; Median (IQR) age: 11.80 (7.70–14.80) years;  Sex: 50.00% male;  Median (IQR) IQ score: 65.20 (54.00–85.00) | Pharmacological: Everolimus | Placebo | Change in full-scale IQ | CBCL total |
| Royston et al. (2024)^40^ | United Kingdom | IG (n=82) CG (n=129) | Population: Preschoolers with moderate to severe intellectual disabilities;  % with ID, global DD, or BIF: 100%; Mean (SD) age: 3.70 (1.00) years;  Sex: 75.00% male | Non-Pharmacological (Psychosocial):  Stepping Stones Triple P | Treatment as Usual:  defined as local access for families to interventions and therapies | Challenging behaviour | CBCL total |

| **Table S3.** *(Continued.)* | | | | | | | | |
| --- | --- | --- | --- | --- | --- | --- | --- | --- |
| **Study** | **Study Location** | **Groups** | **Participant Characteristics** | **Type of Intervention** | **Comparator** | | **Primary Outcome** | **Behaviours that Challenge Measure** |
| Schuiringa et al. (2017)^42^ | The Netherlands | IG (n=80) CG (n=55) | Population: Children with Externalizing Behavior and Mild to Borderline Intellectual Disabilities (MBID);  % with ID, global DD, or BIF: 100%; Mean (SD, range) age: 12.50 (1.99, 9-16) years; Sex: 71.60% male;  Mean (SD) IQ score: 74.20 (10.44) | Non-Pharmacological (Psychosocial): Standing Strong Together (SST) combined with Treatment as Usual | | Treatment as Usual: combination of child behavior management in daycare treatment, parental guidance, and additional individual treatment for children | Externalising behaviour | CBCL total |
| Shapiro et al. (2014)^43^ | United States | Post:  IG (n=41) CG (n=43)  Follow-Up:  IG (n=39)  CG (n=42) | Population: Children with global developmental delay(s), or a diagnosis/condition that elevated future risk for delays;  % with ID, global DD, or BIF: 65%; Mean (SD) age: 19.06 (3.38) months; Sex: 59.00% male | Non-Pharmacological (Psychosocial): Stepping Stones Triple P (SSTP), with the IDEA Part C Early Intervention (EI) services, enhanced by Parent-Provider Partnerships in Child Care (PCAN) training for providers | | Treatment as Usual: the IDEA Part C Early Intervention (EI) services, enhanced by PCAN training alone | Behaviour problems | CBCL total |
| Sutherland et al. (2024)^45^ | United Kingdom | IG (n=30) CG (n=30) | Population: Children with a developmental disability;  % with ID, global DD, or BIF: 65%;  Mean (SD, range) age: 10.03 (1.70, 8–13) years; | Non-Pharmacological (Psychosocial):  Positive Family Connections Intervention | | Waitlist | Family functioning | SDQ subscales:  Internalising, Externalising |
| Tan et al. (2018)^46^ | United States | IG (n=29) CG (n=26) | Population: Children with Angelman Syndrome;  % with ID, global DD, or BIF: 100%; Mean (SD) age: 88.30 (31.50) months; Sex: 54.50% male | Pharmacological: Levodopa | | Placebo | Behavioural problems and developmental outcomes | ABC subscales: Irritability, Lethargy, Stereotypy, Hyperactivity |

| **Table S3.** *(Continued.)* | | | | | | | | |
| --- | --- | --- | --- | --- | --- | --- | --- | --- |
| **Study** | **Study Location** | **Groups** | **Participant Characteristics** | **Type of Intervention** | **Comparator** | | **Primary Outcome** | **Behaviours that Challenge Measure** |
| te Brinke et al. (2022)^47^ | The Netherlands | IG (n=20)  CG (n=22) | Population: Adolescents with externalising problems and Intellectual Disabilities;  % with ID, global DD, or BIF: 100%; Mean (SD) age: 15.52 (1.45) years;  Sex: 50.00% male  Mean (SD) IQ score: 75.66 (7.40) | Non-Pharmacological (Psychosocial):  Cognitive Behavior Therapy Targeting Emotion Regulation (Think Cool then Act Cool emotion regulation training) | | Different Sequence:  Cognitive Behavioral Therapy Targeting Emotion Regulation (Act Cool then Think Cool emotion regulation training) | Externalising problems | Dutch version of the CBCL subscale: Externalising Problems |
| Zhu et al. (2017)^50^ | China | IG (n=40)  CG (n=37) | Population: Children with Global Developmental Delay;  % with ID, global DD, or BIF: 100%; Mean (SD) age: 22.00 (5.50) months;  Sex: 53.30% male | Non-Pharmacological (Diet): Ketogenic diet | | Treatment as Usual:  Comprehensive rehabilitation training | Neurobehavioral development | CBCL total |
| Zwanenburg et al. (2016)^51^ | The Netherlands | IG (n=9)  CG-1 (n=8)  CG-2 (n=8) | Population: Dutch children with Phelan-McDermid syndrome;  % with ID, global DD, or BIF: 100%; Mean (SD, range) age: 82.50 (47.80, 13–189) months; Sex: 24% male | Pharmacological: Intranasal insulin | | Placebo | Level of general developmental functioning | CBCL subscales: Anxiety, Withdrawn, Attention, Aggression |

*Note*. IG = intervention group, CG = control group

| **Table S4.** *Summary of Results of the Included Randomised Controlled Trials (n=20)* | |
| --- | --- |
| **Study** | **Summary of Results** |
| Acosta et al. (2019)^2^ | Pre-intervention Mean (SD): IG=62.75 (13.6), CG=62.95 (13.9)  Post-intervention Mean (SD): IG=53.7 (4.9), CG=61.4 (14.1) |
| Berry-Kravis et al. (2016)^7^ | Change Mean (SE):  ABC-Irritability: IG 25mg=−1.9 (1.41); IG 50mg=−0.6 (1.52); IG 100mg=1.5 (1.28); CG=−1.2 (1.22)  ABC-Lethargy/withdrawal: IG 25mg=−0.9 (0.83); IG 50mg=−1.0 (0.89); IG 100mg=0.5 (0.75); CG=−1.3 (0.71)  ABC-Stereotypic behaviour: IG 25mg=−1.6 (0.52); IG 50mg=−0.4 (0.55); IG 100mg=0.0 (0.47); CG=−0.8 (0.45)  ABC-Hyperactivity: IG 25mg=−1.9 (0.79); IG 50mg=0.3 (0.84); IG 100mg=−0.6 (0.71); CG=−1.0 (0.67)  ABC-Inappropriate speech: IG 25mg=−0.9 (0.38); IG 50mg=0.0 (0.4); IG 100mg=0.1 (0.34); CG=−0.8 (0.33)  ABC-Social avoidance: IG 25mg=−0.6 (0.35); IG 50mg=−0.9 (0.38); IG 100g=−0.6 (0.32); CG=−1.1 (0.31) |
| Berry-Kravis et al. (2017)^8^ | ABC-Social Avoidance: Pre-intervention Mean (SE): IG 5mg BID=6.9 (2.84); IG 10mg BID=6.9 (3.03); IG 10mg TID=6.4 (2.92); CG=6.9 (3.49)  Post-intervention Mean (SE): IG 5mg BID=3.8 (2.46); IG 10mg BID=4.1 (2.85)(I 10mg BID); IG 10mg TID=2.9 (2.72); CG=4.2 (3.24)  ABC-Irritability: Pre-intervention Mean (SE): IG 5mg BID=29.7 (11.16); IG 10mg BID=28.0 (10.81); IG 10mg TID=30.4 (9.53); CG=27.8 (12.91)  Post-intervention Mean (SE): IG 5mg BID=21.7 (9.71); IG 10mg BID=19.5 (11.81), IG 10mg TID=21.6 (10.95); CG=22.4 (14.53)  ABC-Hyperactivity: Pre-intervention Mean (SE): IG 5mg BID=21.6 (6.93); IG 10mg BID=19.4 (6.58); IG 10mg TID=21.9 (5.29); CG=19.6 (7.82)  Post-intervention Mean (SE): IG 5mg BID=17.2 (7.31); IG 10mg BID=15.1 (7.57); IG 10mg TID=15.9 (7.22); CG=15.7 (8.28)  ABC-Stereotypic behaviour: Pre-intervention Mean (SE): IG 5mg BID=11.0 (4.81); IG 10mg BID=9.6 (4.77); IG 10mg TID=11.2 (5.01); CG=9.3 (5.81)  Post-intervention Mean (SE): IG 5mg BID=7.3 (4.26); IG 10mg BID=6.7 (4.64); IG 10mg TID=7.9 (5.12); CG=6.3 (5.52)  ABC-Lethargy: Pre-intervention Mean (SE): IG 5mg BID=12.8 (6.05); IG 10mg BID=13.3 (5.40); IG 10mg TID=13.2 (5.08); CG=11.6 (6.48)  Post-intervention Mean (SE): IG 5mg BID=7.4 (5.03); IG 10mg BID=8.0 (5.09); IG 10mg TID=7.2 (5.94); CG=7.3 (4.99)  ABC-Inappropriate speech: Pre-intervention Mean (SE): IG 5mg BID=7.2 (3.51); IG 10mg BID=7.5 (3.52); IG 10mg TID=7.9 (3.54); CG=6.2 (3.86)  Post-intervention Mean (SE): IG 5mg BID=5.3 (3.48); IG 10mg BID=5.7 (3.93); IG 10mg TID=6.1 (3.43); CG=4.9 (3.76) |
| Berry-Kravis et al. (2022)^9^ | ABC-Social Avoidance: Change Mean: IG=-2.68; CG=-2.29  Treatment Difference (SE): -0.39(0.39), p=.321  ABC-Irritability: Change Mean: IG=-5.88; CG=-4.14  Treatment Difference (SE): -1.74(1.20), p=.149  ABC-Lethargy: Change Mean: IG=-3.50(I); CG=-3.14  Treatment Difference (SE): -0.36(0.70), p=.607 |
| Blasi et al. (2020)^10^ | CBCL Total: Pre-intervention Mean (SD): IG=64.87 (13.06); CG=53.00 (11.51)  Post-intervention Mean (SD): IG=53.60 (10.60); CG=55.33 (9.60)  CBCL Internalising: Pre-intervention Mean (SD): IG=64.00 (12.44); CG=52.83 (11.52)  Post-intervention Mean (SD): IG=56.73 (11.96); CG=62.25 (5.01)  CBCL Externalising: Pre-intervention Mean (SD): IG=56.00 (11.63); CG=49.08 (9.92)  Post-intervention Mean (SD): IG=49.13 (8.81); CG=55.33 (9.60) |

| **Table S4.** *(Continued.)* | |
| --- | --- |
| **Study** | **Summary of Results** |
| Efron et al. (2020)^17^ | ABC-Irritability: Pre-intervention Mean (SD): IG=26.3 (4.2); CG=21.3 (14.6)  Post-intervention Mean (SD): IG=14.3 (2.1); CG=18.8 (14.2)  ABC-Social withdrawal: Pre-intervention Mean (SD): IG=14.0 (6.6); CG=6.0 (5.0)  Post-intervention Mean (SD): IG=5.3 (3.2); CG=5.3 (3.0)  ABC-Stereotypic behaviour: Pre-intervention Mean (SD): IG=10.3 (8.1); CG=3.3 (4.6)  Post-intervention Mean (SD): IG=5.0 (1.0); CG=1.5 (1.3)  ABC-Hyperactivity/non-compliance: Pre-intervention Mean (SD): IG=28.3 (4.0); CG=28.3 (15.2)  Post-intervention Mean (SD): IG=11.3 (5.7); CG=24.0 (12.8)  ABC-Inappropriate speech: Pre-intervention Mean (SD): IG=3.0 (2.7); CG=2.3 (4.5)  Post-intervention Mean (SD): IG=1.7 (1.5); CG=0.8 (1.0) |
| Fastman et al. (2021)^21^ | ABC-Irritability: Pre-intervention Mean (SD): IG=9.57 (8.75); CG=10.33 (5.24)  Post-intervention Mean (SD): IG=7.86 (8.31); CG=5.33 (3.12)  ABC-Social withdrawal: Pre-intervention Mean (SD): IG=14.29 (3.59); CG=19.67 (6.34)  Post-intervention Mean (SD): IG=11.86 (6.04); CG=12.22 (6.24)  ABC-Stereotypy: Pre-intervention Mean (SD): IG=6.29 (3.04); CG=9.22 (4.24)  Post-intervention Mean (SD): IG=6.86 (2.48); CG=7 (4.8)  ABC-Hyperactivity: Pre-intervention Mean (SD): IG=21.71 (12.88); CG=29.67 (9.39)  Post-intervention Mean (SD): IG=19 (10.74); CG=21.56 (12.42)  ABC-Inappropriate speech: Pre-intervention Mean (SD): IG=3 (4.04); CG=4 (3.64)  Post-intervention Mean (SD): IG=2.29 (2.36); CG=2.33 (3.16) |
| Johnson et al. (2019)^26^ | T(wald) = -0.29; p = .77; d = -0.27 at Week 20 |
| Kostulski et al. (2021)^27^ | Pre-intervention Mean (SD): IG=0.78 (0.19); CG=0.75 (0.17)  Post-intervention Mean (SD): IG=0.61 (0.21); CG=0.72 (0.15) |
| Lo et al. (2015)^28^ | N/A |
| Navarro et al. (2015)^33^ | ABC-Hyperactivity: Pre-intervention Mean: IG=21.6; CG=24.5  Post-intervention Mean: IG=21.0; CG=26.5  ABC-Irritability: Pre-intervention Mean: IG=19.70; CG=10.50  Post-intervention Mean: IG=16.00; CG=14.50  CBCL-Inattention: Pre-intervention Mean (T-score): IG=9.6 (67.8); CG=9.5 (68.75)  Post-intervention Mean (T-score): IG=8.7 (67); CG=8.25 (65.5) |
| Overwater et al. (2019)^36^ | Treatment effect everolimus vs placebo (95% CI)=8.1 (−5.0 to 21.1) |

| **Table S4.** *(Continued.)* | |
| --- | --- |
| **Study** | **Summary of Results** |
| Royston et al. (2024)^40^ | Pre-intervention Mean (SD): IG=96.1 (24.8); CG=93.3 (28.3)  Post-intervention Mean (SD): IG=90.0 (31.2); CG=91.0 (30.1) |
| Schuiringa et al. (2017)^42^ | Pre-intervention Mean (SD): IG=67.74 (7.00); CG=67.09 (8.13)  Post-intervention Mean (SD): IG=63.80 (7.65); CG=64.40 (9.17) |
| Shapiro et al. (2014)^43^ | Study 1 CBCL Total: Pre-intervention Mean (SD): IG=53.56 (8.54); CG=51.00 (10.67)  Post-intervention Mean (SD): IG=49.82 (10.04); CG=49.91 (9.05)  Study 1 CBCL Internalising: Pre-intervention Mean (SD): IG=51.44 (8.29); CG=50.33 (10.05)  Post-intervention Mean (SD): IG=56.73(11.96); CG=62.25(5.01)  Study 1 CBCL Externalising: Pre-intervention Mean (SD): IG=53.96 (10.62); CG=51.08 (11.19)  Post-intervention Mean (SD): IG=53.55 (12.83); CG=51.26 (9.31)  Study 2 CBCL Total: Pre-intervention Mean (SD): IG=51.05 (10.51); CG=51.70 (9.41)  Post-intervention Mean (SD): IG=51.42 (10.09); CG=54.9 (11.88)  Study 2 CBCL Internalising: Pre-intervention Mean (SD): IG=48.95 (8.76); CG=49.20 (8.55)  Post-intervention Mean (SD): IG=48.16 (9.81); CG=51.25 (9.28)  Study 2 CBCL Externalising: Pre-intervention Mean (SD): IG=51.00 (10.93); CG=50.80 (11.52)  Post-intervention Mean (SD): IG=52.42 (9.48); CG=54.8 (12.08) |
| Sutherland et al. (2024)^45^ | SDQ Internalising: Pre-intervention Mean (SD): IG=11.20 (4.30); CG=10.77 (4.01)  Post-intervention Mean (SD): IG=9.75 (3.96); CG=10.33 (4.07)  SDQ Externalising: Pre-intervention Mean (SD): IG=11.07 (3.71); CG=11.73 (2.57)  Post-intervention Mean (SD): IG=9.93 (3.66); CG=10.82 (2.84) |
| Tan et al. (2018)^46^ | ABC-Irritability: Pre-intervention Mean (SD): IG=9.1 (9.3); CG=4.8 (4.6)  Post-intervention Mean (SD): IG=7.3 (8.0); CG=6.6 (7.1)  ABC-Lethargy: Pre-intervention Mean (SD): IG=4.7 (4.6); CG=2.7 (3.3)  Post-intervention Mean (SD): IG=4.5 (3.9); CG=5.2 (8.4)  ABC-Stereotypy: Pre-intervention Mean (SD): IG=5.1 (3.7); CG=3.0 (3.6)  Post-intervention Mean (SD): IG=3.5 (3.7); CG=3.6 (3.9)  ABC-Hyperactivity: Pre-intervention Mean (SD): IG=19.5 (12.2); CG=15.5 (10.8)  Post-intervention Mean (SD): IG=18.2 (9.9); CG=17.0 (10.4) |
| te Brinke et al. (2022)^47^ | Pre-intervention Mean (SD): IG=0.85 (0.28), CG=0.85 (0.28)  Post-intervention Mean (SD): IG=0.53 (0.28); CG=0.74 (0.31) |
| Zhu et al. (2017)^50^ | Pre-intervention Mean (SD): IG=68 (3); CG=67 (3)  Post-intervention Mean (SD): IG=66 (7); CG=68 (7) |

| **Table S4.** *(Continued.)* | |
| --- | --- |
| **Study** | **Summary of Results** |
| Zwanenburg et al. (2016)^51^ | The coefficient (C) provides an estimate of the change in t-score in points per 6 months:  CBCL-Anxiety: IG=0.36; CG=-0.80  CBCL-Withdrawn: IG=1.34; CG=-3.15  CBCL-Attention: IG=-1.17; CG=0.04  CBCL-Aggression: IG=0.23; CG=-1.18 |

*Note*. IG = intervention group, CG = control group, BID = bis in die (twice a day), TID = ter in die (three times a day)

*Note*. scores derived from each study were not transformed and were in the original scale of the measure
